# Rapid non-invasive prenatal screening test for trisomy 21 based on digital droplet PCR

**DOI:** 10.1038/s41598-023-50330-x

**Published:** 2023-12-22

**Authors:** Soňa Laššáková, Pavel Šenkyřík, Eva Pazourková, Aleš Hořínek, Pavel Calda, Miroslav Břešťák, Kamila Světnicová, Pavel Neužil, Marie Korabečná

**Affiliations:** 1 Institute of Biology and Medical Genetics, First Faculty of Medicine, Charles University and General University Hospital in Prague, Albertov 4, 128 00 Prague, Czech Republic; 2 Department of Nephrology, First Faculty of Medicine, Charles University and General University Hospital in Prague, U Nemocnice 499/2, 128 08 Prague 2, Czech Republic; 3 3rd Department of Internal Medicine, First Faculty of Medicine, Charles University and General University Hospital in Prague, U Nemocnice 1, 128 08 Prague 1, Czech Republic; 4 Department of Gynaecology, Obstetrics and Neonatology, First Faculty of Medicine, Charles University and General University Hospital in Prague, Apolinarska 18, 128 51 Prague, Czech Republic; 5 Prenatal Diagnosis Center ProfiG2 S.R.O., Vajgarská, 1141 Prague, Czech Republic; 6 GENvia, Karlovo náměstí 7, 128 00 Prague 2, Czech Republic; 7 Department of Microsystem Engineering, School of Mechanical Engineering, Northwestern Polytechnical University, 127 West Youyi Road, Xi’an, 710072 Shaanxi People’s Republic of China; 8 Department of Laboratory Medicine, Faculty of Health Care and Social Work, University of Trnava in Trnava, Universitne Namestie 1, 918 43 Trnava, Slovak Republic

**Keywords:** Biochemistry, Genetics, Molecular medicine

## Abstract

Non-invasive prenatal tests for the detection of fetal aneuploidies are predominantly based on the analysis of cell-free DNA (cfDNA) from the plasma of pregnant women by next-generation sequencing. The development of alternative tests for routine genetic laboratories is therefore desirable. Multiplex digital droplet PCR was used to detect 16 amplicons from chromosome 21 and 16 amplicons from chromosome 18 as the reference. Two fluorescently labeled lock nucleic acid probes were used for the detection of reaction products. The required accuracy was achieved by examining 12 chips from each patient using Stilla technology. The plasma cfDNA of 26 pregnant women with euploid pregnancies and 16 plasma samples from pregnancies with trisomy 21 were analyzed to determine the cutoff value for sample classification. The test was validated in a blind study on 30 plasma samples from pregnant patients with a risk for trisomy 21 ranging from 1:4 to 1:801. The results were in complete agreement with the results of the invasive diagnostic procedure (sensitivity, specificity, PPV, and NPV of 100%). Low cost, and speed of analysis make it a potential screening method for implementation into the clinical workflow to support the combined biochemical and ultrasound results indicating a high risk for trisomy 21.

## Introduction

Trisomy 21 (T21), known as Down syndrome (DS), is the most common chromosomal abnormality occurring in humans. It is caused by trisomy of chromosome 21 (Chr21) or a part of it^1,2^. Its complex phenotype is a result of a dosage imbalance of genes located in human Chr21 that affects multiple body systems^3,4^. The incidence of T21 varies in different populations, for example, from 1 in 319 to 1 in 1000 live births^2,4^. In the Czech Republic, where we obtained our clinical samples, it is 1 in 393^5^.

In 1968, the first antenatal diagnosis of T21 was made^6^. Since then, screening for T21 has been introduced into medical practice. It was supposed to limit diagnostic procedures (amniocentesis [AMC] or chorionic villus sampling [CVS])^3^, which are considered "the gold standard” for the final diagnosis of fetal aneuploidies. However, these invasive procedures can lead to a risk of iatrogenic miscarriage of up to 1% and other complications^7^.

Since then, screening approaches have involved maternal age and a combination of assessing maternal serum biochemical analytes and prenatal ultrasonography. First-trimester screening (combined test) has a detection rate (DR) of 82–87% and a 2–3% positive predictive value (PPV)^8–10^. Second-trimester screening has a DR of approximately 69–81% and a 2% PPV^9,10^. A combination of the first and second-trimester screening results provides a higher DR of approximately 88–96%)^9^. However, PPV is again only between 3 and 5%^11^.

There was continued interest in more precise and non-invasive prenatal testing (NIPT) for fetal chromosomal aneuploidies. A breakthrough came in 1997 when the presence of fetal nucleic acids in maternal circulation was reported as cell-free fetal DNA (cffDNA)^12^. Because of the low fetal fraction (FF) in total cell-free DNA (cfDNA) (on average 10–15% between the tenth and twentieth gestational week)^10,13^, it took a few years to develop the methodology based on next generation sequencing (NGS) for accurately measuring a dose of cffDNA from extra Chr21 in the plasma of pregnant women^14^.

The cfDNA screening by NGS has excellent sensitivity and specificity^9,11^, but the procedure is time-consuming, complex, and expensive^15^. This makes it difficult to implement NGS into widespread clinical routine laboratory tests. To overcome this disadvantage of NGS, digital droplet PCR (ddPCR)^16^ could be utilized as an alternative approach. Compared to NGS, this method is more sensitive, requires less labor and time (only 2–3 h), and the cost to run the test is also lower^7,15^.

Recently, a few publications proposed a similar strategy for better utilization of ddPCR in NIPT, which is based on high-level polymerase chain reaction (PCR) multiplexing with probes labeled only with two fluorophores—one fluorophore for Chr21 and the second one for the reference chromosome^7,17,18^. This approach enables the retrieval of a greater number of positive PCR reactions from samples with low concentrations of cfDNA originating from both the mother and fetus. This increases the statistical confidence of the results.

We used sequences of primers and probes published earlier^18^ for application on another dPCR platform, however, it was necessary to modify the original published set. Unlike the previously published report^18^, we confirmed the results of our validation study not with a screening test at the cfDNA level, but with the result of invasive prenatal diagnostics.

In this study, we have described the ddPCR method, which allows distinguishing between euploid and trisomic pregnancies using a multiplex of 32 primer pairs and universal locked nucleic acid (LNA) probes. In order to ensure the highest accuracy, isolated cfDNA samples were concentrated, and the results of 12 tests were merged to achieve ≈ 240,000 partitions for each analyzed sample. Our approach makes the method fast (one working day), low-cost, and results in a high PPV. Confirmation of fetal karyotypes for all pregnancies was done either cytogenetically or by QF-PCR (quantitative fluorescence PCR) after an invasive procedure. Our assay was evaluated in pilot phase, where 42 plasma samples (26 euploid and 16 T21 samples) were tested. Then it was validated in validation (blind) phase, where 30 plasma samples were tested (26 euploid and 4 T21 samples).

## Results

### Multiplex ddPCR optimization

In the first part of method optimization (using the control genomic DNA), all primers were tested in single target reactions, and the amplicons were detected with corresponding probes. In the second part, multiplexing was developed.

**Figure 1:**
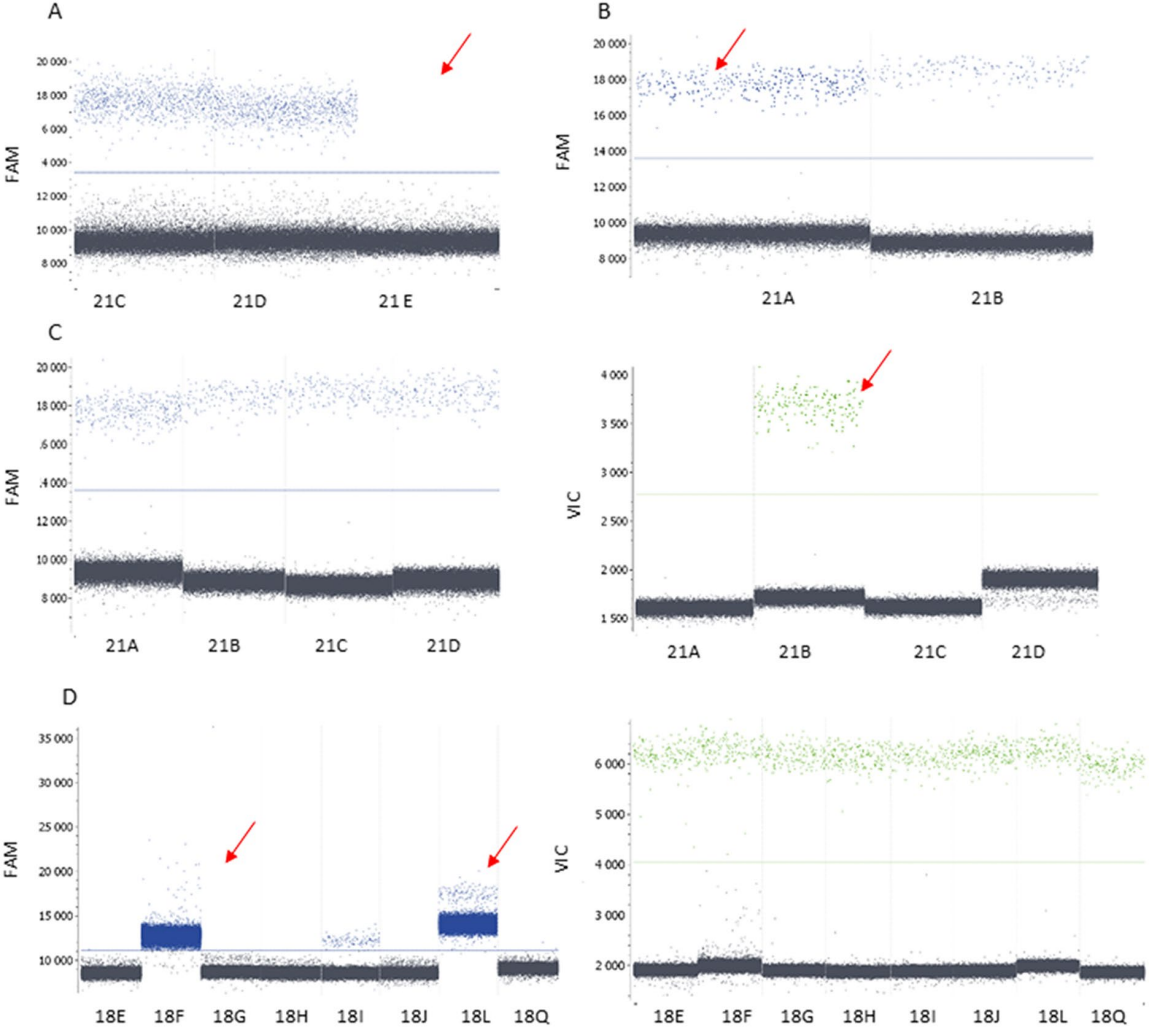
Selection of primer pairs for multiplexing—demonstration of selected results leading to the primer pair exclusion. (**A**) No amplification of a target. (**B**) Higher amplification with one primer pair. (**C**) Detection of an amplicon by both probes. (**D**) Successful amplification but elevated fluorescence of negative droplets in the second channel.

**Figure 2:**
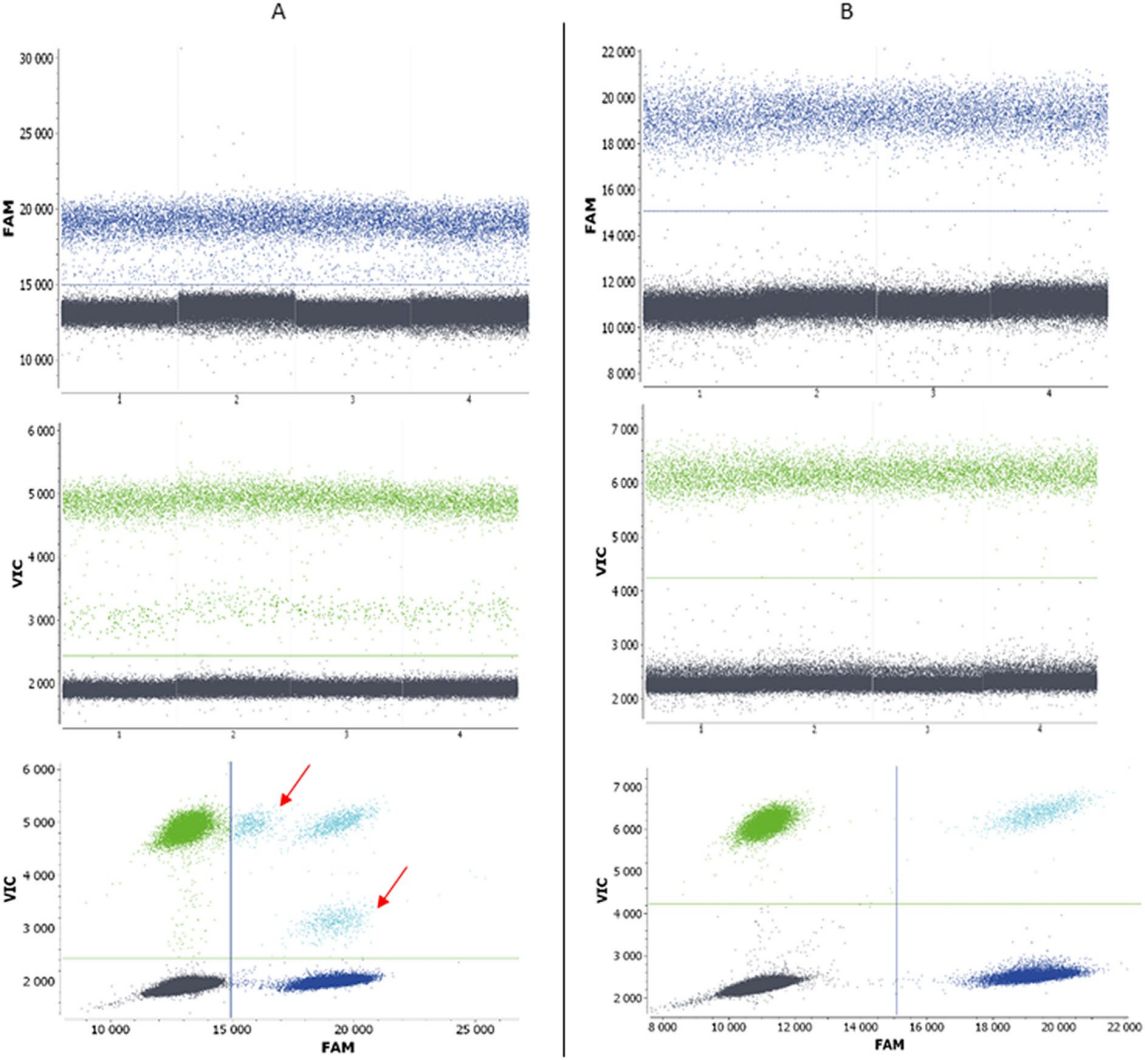
Results of multiplex ddPCR optimization. (**A**) Multiplex with 18 primer pairs for each chromosome, including two pairs of primers (21B and 18I) whose amplicons were detected by both probes (marked by arrows) and primer pairs that elevated the fluorescence of negative droplets. (**B**) Performance of multiplex ddPCR after exclusion of primer pairs due to the reasons mentioned above.

Some of the primers reported earlier^18^ were excluded. There were four reasons for the exclusion of primer pairs: (1) there was no amplification of a target, (2) amplification with the primer pair led to a higher amount of product than with the other primer pairs, (3) the same amplicon was detected by both probes, and (4) amplification with the primer pair was successful but elevated the fluorescence of negative droplets in the second channel (Figs. 1, 2). After optimization, the final multiplex was chosen, amplifying 16 amplicons from each chromosome (Supplementary Tables 1, 2, 3 and 4). This multiplex led to the ratio 21/18 equal to 0.9715 for control genomic DNA and 1.4555 for genomic DNA of an individual with T21, thus correctly mirroring the number of Chr 21 in karyotypes.

### Analysis of artificial mixtures

**Figure 3:**
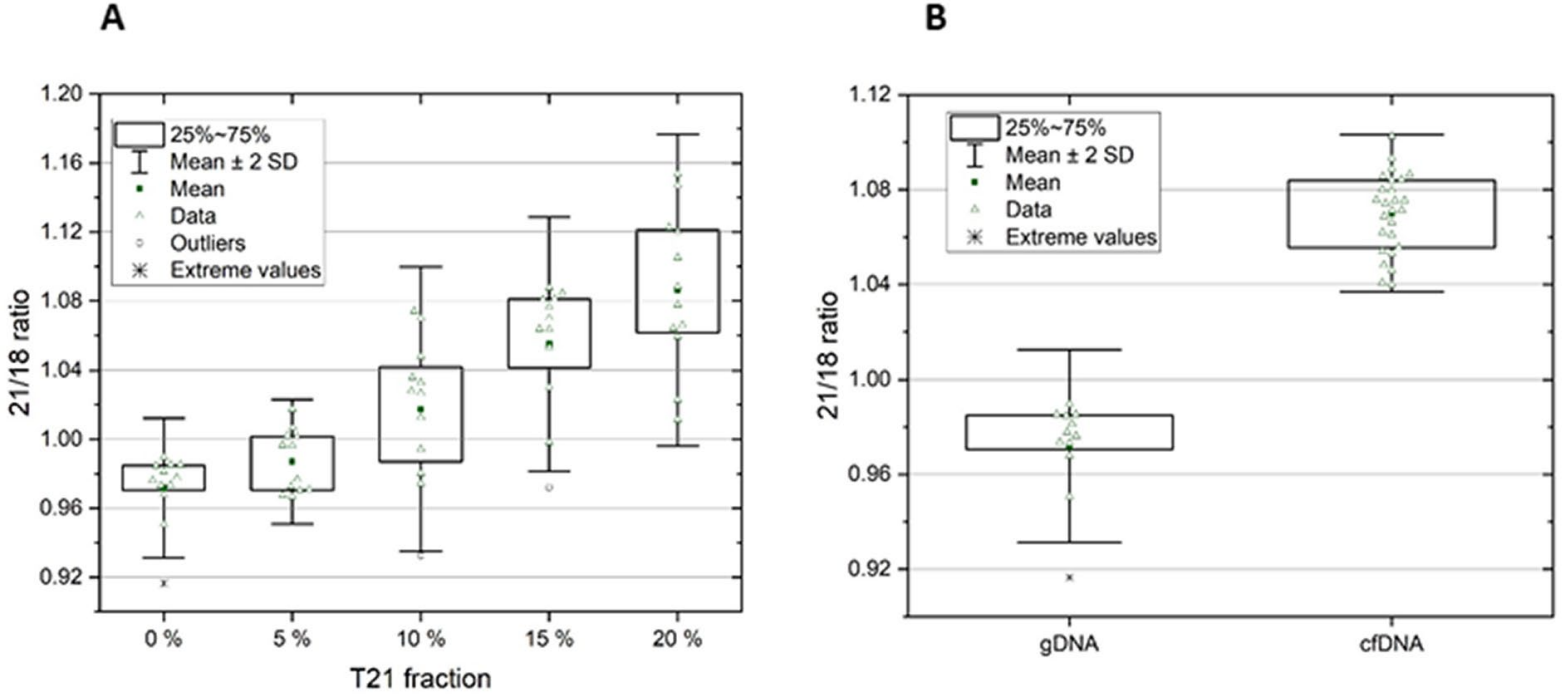
(**A**) Analysis of artificial mixtures of genomic DNAs. (**B**) Comparison of 21/18 ratios obtained from genomic female DNA and cfDNA from 26 euploid pregnancies.

In this part of the study, the methodology was assessed in order to detect a small increase in chromosome ratios in pregnancies with T21 by mimicking plasma DNA from pregnant women. The mixtures reflecting the possible proportions of cffDNA in total cfDNA were prepared by using human female genomic DNA and human T21 genomic DNA^17,19^. We prepared 0.125 ng/µl DNA artificial mixtures containing 20%, 15%, 10%, 5%, and 0% of human T21 genomic DNA. For each mixture, a ddPCR assay was performed in 12 replicates (Table 1, Supplementary Table 5, Fig. 3). The data obtained from all 12 replicates for each mixture were combined, and the uncertainties were calculated using the equation described in the literature^20^. The combination led to a significant decrease in uncertainty (Table 1, Supplementary Table 5). Uncertainties achieved in this experiment with artificial mixtures seemed to be sufficient for the clear distinguishing of the pregnancies with T21 if more than 5% of fetal cfDNA is present in the maternal plasma.

**Table 1:** Ratios 21/18 obtained in the analysis of artificial mixtures; each mixture was analyzed in 12 technical replicates.

Replicate no	0% of T21	Uncertainty for		5% of T21	Uncertainty for		10% of T21	Uncertainty for	
	21/18	Chr 21 (%)	Chr 18 (%)	21/18	Chr 21 (%)	Chr 18 (%)	21/18	Chr 21 (%)	Chr 18 (%)
1	0.9846	4.11	4.08	1.0022	4.47	4.47	0.9801	3.93	3.93
2	0.9735	4.15	4.09	0.9675	4.15	4.09	0.9743	3.93	3.93
3	0.9896	4.09	4.07	0.9667	4.16	4.09	1.0358	3.94	3.94
4	0.9505	4.16	4.06	0.9965	3.97	3.96	0.9327	3.91	3.91
5	0.9853	4.35	4.32	1.0172	4.04	4.08	1.0126	4.00	4.00
6	0.9679	4.25	4.19	0.9724	4.05	4.00	1.0740	4.30	4.30
7	0.9732	4.09	4.04	0.9708	4.11	4.05	1.0329	4.00	4.00
8	0.9778	4.07	4.03	1.0068	4.07	4.08	0.9942	4.00	4.00
9	0.9165	4.37	4.19	0.9965	4.10	4.09	1.0477	4.00	4.00
10	0.9852	4.14	4.11	0.9702	4.06	4.00	1.0279	3.99	3.99
11	0.9813	4.18	4.14	0.9766	4.02	3.97	1.0698	4.02	4.02
12	0.9762	4.10	4.06	1.0013	3.98	3.98	1.0268	3.94	3.94
21/18 values and uncertainty after merging	0.9718	1.21	1.22	0.9871	1.22	1.22	1.0174	1.18	1.19
Theoretical 21/18 ratio	1.000			1.025			1.050

However, mixtures created from genomic DNA and T21 DNA may not fully reflect the conditions in maternal plasma, which is the true source of cfDNA needed for the analysis. It is extremely difficult to perfectly imitate the conditions in maternal plasma. In order to see the difference, the ratios of 21/18 achieved in the samples of pregnant women with euploid fetuses and the ratios obtained from our genomic control DNA were compared (Fig. 3). According to this comparison, higher values of the ratio 21/18 were obtained by examining real plasma samples. This part of the study also clearly demonstrated the importance of combining the results of 12 technical replicates to achieve the uncertainty necessary to distinguish T21 pregnancies.

### Analysis of cfDNA from pregnant patients

**Figure 4:**
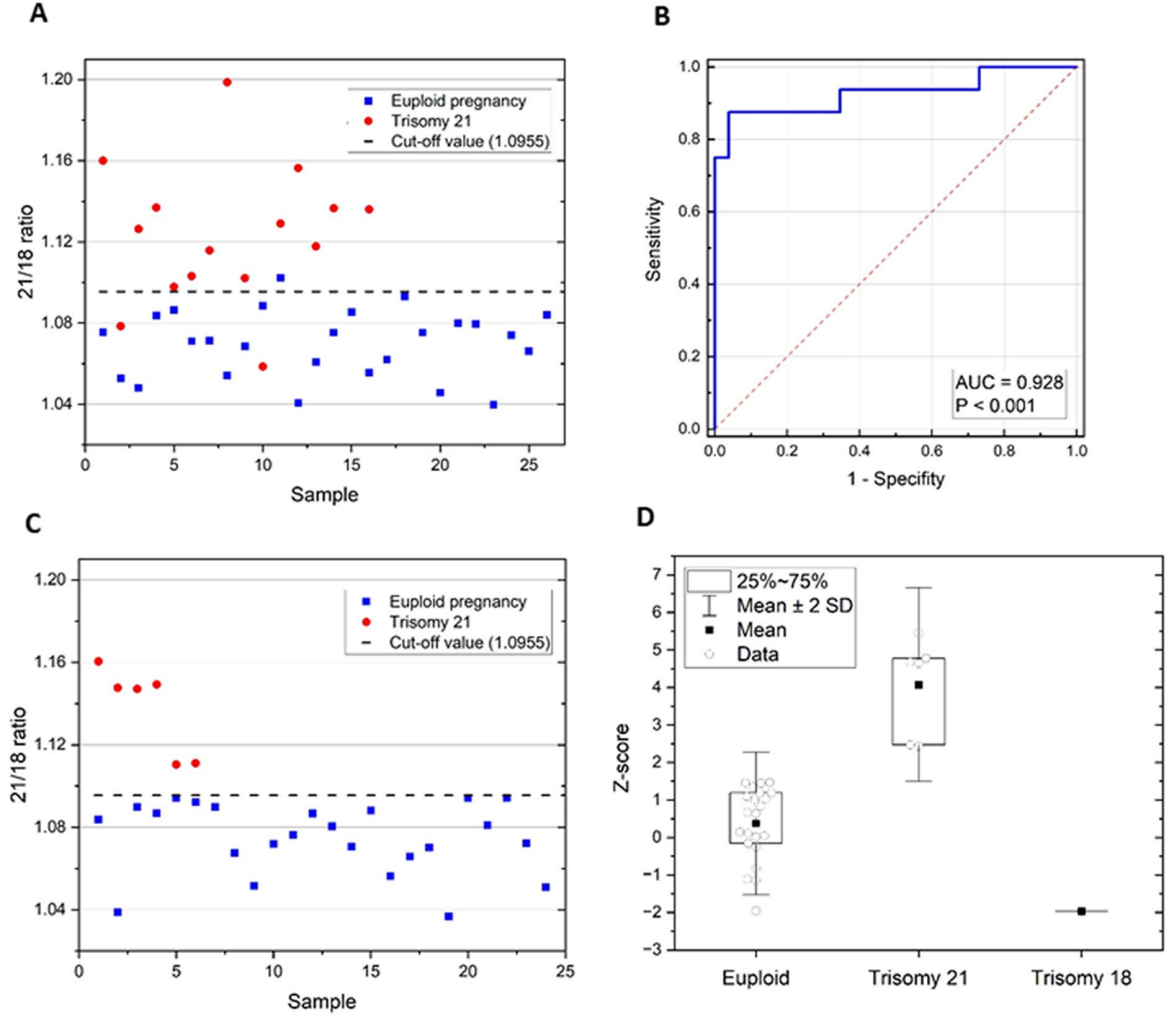
Pilot and validation phase results. (**A**) The pilot phase served for the cutoff value determination (the dashed line represents R = 1.0955 set according to the ROC curve) to differentiate the euploid and T21 pregnancies. (**B**) The ROC curve is based on the results of the pilot phase. (**C**) The validation phase results were interpreted using the cutoff value set in the pilot phase. (**D**) Validation phase results are presented as Z-score.

In the pilot phase, the cutoff value of the ratio 21/18 for the classification of euploid pregnancies and pregnancies with T21 was established by creating a receiver operating characteristic (ROC) curve from the results of this phase. The cutoff value of 1.0955 was chosen to obtain the maximum sensitivity and specificity possible. The area under the curve was equal to 0.928 with a *p* value < 0.001, indicating that the test has excellent discriminating ability (Fig. 4A, B, Supplementary Tables 6 and 7)^21^. Using this cutoff value, 14 out of 16 pregnancies with T21 were correctly identified in the pilot phase. The test reached 96.2% specificity and 87.5% sensitivity. The PPV was 93.3%, and the NPV was 92.6%.

The test was validated on 30 plasma samples from pregnant patients with a risk for T21 ranging from 1:4 to 1:801. Using the cutoff value set in the previous phase, the status of all examined pregnancies was correctly determined (Fig. 4C, Table 2, Supplementary Table 8). The results of the validation phase were also presented as Z-scores. Euploid and T21 pregnancies were well distinguished (Fig. 4D). Our findings were completely consistent with the findings of subsequent invasive diagnostic procedures, and all test parameters (sensitivity, specificity, PPV, and NPV) reached 100%.

**Table 2:** Information of clinical samples used in the validation phase and the results of multiplex ddPCR.

Sample no	Gestational week	Fetal sex	Maternal age	Nuchal translucency (mm)	Risk of T21	Chr21/Chr18	ddPCR result	CVS/AMC result
1	12 + 6	Female	33	2.4	1:88	1.1605	Trisomy 21	Trisomy 21
2	12 + 4	Male	36	2.6	1:4	1.1476	Trisomy 21	Trisomy 21
3	13 + 1	Male	36	2.3	1:19	1.1471	Trisomy 21	Trisomy 21
4	13 + 6	Female	37	2.5	1:7	1.1493	Trisomy 21	Trisomy 21
5	12 + 5	Male	37	3.23	1:19	1.1105	Trisomy 21	Trisomy 21
6	13 + 2	Male	36	4.0	1:4	1.1111	Trisomy 21	Trisomy 21
7	12 + 4	Female	40	9.5	1:44	1.0388	Trisomy 18	Trisomy 18
8	14 + 1	Male	28	2.8	1:801	1.0838	Euploid	Euploid
9	13 + 0	Male	29	1.6	1:240	1.0898	Euploid	Euploid
10	18 + 0	Male	38	1.7	1:128	1.0869	Euploid	Euploid
11	13 + 0	Male	38	2.1	1:199	1.0941	Euploid	Euploid
12	12 + 3	Male	26	3.3	1:117	1.0924	Euploid	Euploid
13	13 + 2	Female	28	0.9	1:532	1.0898	Euploid	Euploid
14	13 + 0	Male	26	1.5	1:423	1.0675	Euploid	Euploid
15	12 + 0	Female	35	8.6	1:12	1.0516	Euploid	Euploid
16	13 + 0	Male	39	2.5	1:23	1.0720	Euploid	Euploid
17	12 + 3	Female	29	1.37	1:273	1.0764	Euploid	Euploid
18	13 + 5	Male	47	1.2	1:11	1.0867	Euploid	Euploid
19	13 + 6	Female	41	1.2	1:34	1.0806	Euploid	Euploid
20	12 + 5	Female	34	3.5	1:136	1.0707	Euploid	Euploid
21	13 + 3	Male	33	1.2	1:502	1.0881	Euploid	Euploid
22	13 + 0	Male	38	4.0	1:27	1.0563	Euploid	Euploid
23	12 + 5	Female	30	1.29	1:64	1.0658	Euploid	Euploid
24	14 + 5	Female	32	1.8	1:44	1.0702	Euploid	Euploid
25	12 + 2	Male	37	1.6	1:121	1.0368	Euploid	Euploid
26	13 + 4	Female	37	1.13	1:254	1.0942	Euploid	Euploid
27	12 + 6	Male	39	2.4	1:85	1.0810	Euploid	Euploid
28	12 + 5	Female	38	1.7	1:92	1.0944	Euploid	Euploid
29	15 + 4	Female	39	3.7	1:31	1.0723	Euploid	Euploid
30	13 + 0	Female	39	3.1	1:66	1.0510	Euploid	Euploid

## Discussion

In this study, we performed a modification of the multiplex dPCR designed by Tan et al.^18^ and its adaptation to the Stilla ddPCR platform to distinguish between a euploid pregnancy and a T21 pregnancy.

It was crucial to reach the highest possible number of positive droplets and to achieve the lowest possible uncertainty. For this reason, a sample DNA concentration step was incorporated into the workflow, a multiplex of 16 primer pairs for each chromosome was used, and the merging of 12 tests for one sample was performed.

In the pilot phase of the study, 26 plasma samples of euploid pregnancies and 16 plasma samples with T21 were tested. The cutoff value was determined using the 21/18 ratio values. (Fig. 4). This value was used in the validation phase (blind part of the study) to identify the pregnancies with T21 among patients with a high risk of T21. As part of the first- or second-trimester screening, the risk was calculated using biochemical markers, sonographic examination, and maternal age.

According to our workflow, all affected pregnancies (6 out of 6) in this small cohort were correctly detected (Fig. 4, Table 2). These samples can be divided into two groups. The first group of four samples achieved a relatively high value of the 21/18 ratio and could be easily distinguished from the euploid pregnancies. In contrast, the second group of two samples obtained the value of the 21/18 ratio very close to the cutoff value. The reasons for this difference can be split into two categories.

The first one is the technical point of view. The workflow of the assay was the same for both groups. However, there was a significant time gap between them. As a result, a different batch of mix was used for analysis, and a slight difference in its content may have influenced the 21/18 ratio. The second one is the biological viewpoint, namely the FF of cfDNA. Despite the high number of positive droplets and high accuracy of the assay, FF plays a crucial role in assay reliability. The minimum FF for statistical confidence is typically between 2 and 4% depending on the applied assay. Many biological factors can influence the maternal and placental cfDNA contribution. For example, high maternal weight, autoimmune disease of the mother, parity, or maternal age can all decrease FF^13^.

Low FF can cause the result to enter a gray zone, which can lead to test failure or a “no call” result^13^. In the sequencing-based NIPT method, the gray zone is a special Z-score range in which it is impossible to decide whether the result is positive or negative with high confidence^22^. If the cutoff value of the Z-score (gray zone) was calculated for our assay by using the U-test^23^, it would be 3.73. It would mean that the fetus has a high risk of T21 when the Z-score is over 3.73. The gray zone can also be defined by a lower limit as a *p* value of the U-test. In our case, the lower limit would be 1.96, with a level of confidence of 95%. According to these limits, four samples would be correctly marked as T21, while two samples would fall into the gray zone.

For the gray zone cases, the American College of Obstetricians and Gynecologists recommends that patients be offered a comprehensive ultrasound examination and diagnostic testing because of an increased risk of aneuploidy^9^. Zhang et al. suggested retesting the cases placed in the gray zone starting with DNA extraction to reclassify them into positive or negative^23^.

To avoid "no call" results in advance, a protocol for the enrichment of FF based on its smaller fragment size can be used^7,13^. Another option is to subject samples to quality control in order to detect FF. Unfortunately, currently used methods for measuring FF (based on Y-chromosome, sequencing, or methylation differences) are not standardized and vary considerably. They are not directly comparable and have large variability in reported FF values^13^. According to Yu et al.^24^, a new approach, fragmentomics, which uses DNA fragment size as a diagnostic parameter, maybe a solution for determining FF level.

In the validation phase, a patient with trisomy 18 (T18) in her fetus (patient no. 7 in Table 2) was also detected. The patient was involved in the study due to the high risk of T21, but the value of the ratio 21/18 received after ddPCR analysis was below the twenty-fifth percentile of healthy pregnancies (Table 2, Fig. 4). This result indicated the opposite application of the methodology, namely the detection of T18. However, before using the assay for this purpose, it is necessary to establish cutoff values similar to how it was carried out for T21 in the pilot phase. Proof of that is another patient with a similar value of the ratio 21/18 (patient no. 25 in Table 2) who was carrying a euploid pregnancy.

When comparing the results of the pilot and validation phases, differences in sensitivity and specificity can be seen. While there are two false-negative results and one false-positive result in the pilot phase, none of these are present in the validation phase. This can be caused by a low number of samples in the validation phase or by the different freshness of the samples. Several days after blood collection, plasma samples that were used in the validation phase were processed. However, samples that were used for the pilot phase were stored at − 20 °C for a few years. This could affect the quality and quantity of cfDNA and be reflected in the final result^25^.

In this pilot study, we demonstrated the high potential of the developed methodology on a set of 30 patients in the blind part of the study. All the parameters (sensitivity, specificity, PPV, and NPV) proved to be nearly optimal (Fig. 4). In addition, the low cost and speed of analysis predetermine this method for implementation into the clinical workflow as a screening alternative additionally offered to anxious patients at high risk for T21 before the invasive procedure. As part of our study, a cut-off value was set, the functioning of which is not influenced by the result of other screening examinations, so it could be used also for testing pregnancies with low biochemical screening risk. However, for further refinement, a larger number of pregnant women must be examined.

## Material and methods

### Material

The study used three different types of samples: human female genomic DNA, human T21 genomic DNA, and plasma samples.

The human female genomic DNA (Promega G1521) is commercially available with an indicated concentration of 165 ng/µl and is stored in TE buffer. The human T21 genomic DNA with a concentration of 130 ng/µl was obtained during prenatal diagnostics using cultivated amniocytes. T21 was confirmed by classical cytogenetic analysis (karyotyping). For extraction of the genomic DNA, the QIAmp DNA Mini Kit (QIAGEN, Germany) was used. It was diluted in 100 µl of AE buffer and stored at − 20 °C. These two types of DNA were used for method optimization.

Plasma samples were obtained from pregnant women and collected in cooperation with the Department of Obstetrics, Gynecology and Neonatology of the First Faculty of Medicine of Charles University and the General University Hospital in Prague, and the private company GENvia. A total of 72 plasma samples were divided into three groups: the control group (26 samples), the T21 group (16 samples), and the validation phase group (30 samples). The criteria for selecting samples for the control and T21 groups were the age of gestation (from 8 to 18 weeks) and confirmation of fetal karyotype according to the group. The criteria for selecting samples for the validation phase were age of gestation, a positive result of first-trimester screening, or a second-trimester screening with a high risk of T21 indicating an invasive diagnostic procedure. Fetal karyotypes were confirmed cytogenetically or by quantitative fluorescence PCR after an invasive procedure for all pregnancies.

Plasma samples were used to establish a threshold for distinguishing between the control group and the T21 group. The results of invasive diagnostics for the validation phase were not known to the researchers.

The median gestational week for the control group was 10 (range was from the eighth to the thirteenth week); for the T21 group, it was 14 (range was from the twelfth to the eighteenth week); and for the validation group, it was 13 (range was from the thirteenth to the eighteenth week).

All patients provided written informed consent to participate in the study. The study was conducted in accordance with the Declaration of Helsinki and approved by the Ethical Committee of the First Faculty of Medicine of Charles University and the General University Hospital in Prague.

### Cell-free DNA isolation from plasma samples

For the pilot phase, peripheral blood samples were collected into Vacutainer tubes with ethylenediaminetetraacetic acid, and for the validation phase, into cell-free DNA BCT tubes (Streck). The samples were handled and processed in accordance with the tube manufacturer's instructions. A two-step centrifugation protocol was created to obtain plasma from peripheral blood samples: at 2600*g* for 10 min at 10 °C and at 14,500*g* for 10 min at room temperature. Afterward, plasma samples were stored at – 20 °C.

Cell-free DNA was extracted from 2 ml of plasma (each milliliter was isolated separately) by MagNA Pure Compact System with MagNA Pure Compact Nucleic Acid Isolation Kit I—Large Volume (Roche Diagnostics, Germany) in accordance with the manufacturer's instructions. The cfDNA isolated from each ml of plasma was eluted in 50 µl of supplied elution buffer into Eppendorf tubes (total volume for 2 ml was 100 µl). The purified cfDNA was immediately processed.

Afterward, each sample in the Eppendorf tubes was placed into an IR MICRO-CENVAC NB-503CIR concentrator (N-BIOTEC, Korea) at 1700 RPM at 37 °C for 11 min. The sample was concentrated from 100 to 80 µl.

### Preparation of pre-reactions for ddPCR

For the detection of cfDNA from Chr18 and Chr21, we chose a set of 16 primer pairs for each chromosome. They were selected out of 20 primer pairs used for the amplification of different sequences localized on Chr18 and Chr21 described by Tan et al.^18^. All of them were targeted into conserved regions that did not coincide with copy number variants and common single-nucleotide polymorphisms^18^.

Our selection was based on data from the optimization of the ddPCR reaction (Supplementary Tables 1, 2, 3 and 4). For the detection of positive droplets, LNA probes published by Tan et al.^18^ were used. LNA probes have a higher affinity to the complementary DNA than other types of probes due to the modification in oligonucleotide bases (methylene bridge bond between 2′ oxygen and 4′ carbon of the pentose ring). It increases both duplex stability and mismatch discrimination^26^. The LNA probe for 16 primer pairs for Chr21 was FAM-labeled, and the LNA probe for 16 primer pairs for Chr18 was VIC-labeled.

At the beginning of the ddPCR workflow, a pre-reaction mixture of 25 µl was prepared. The mixture consisted of 10 µl of PerfeCTa Multiplex qPCR ToughMix (Quanta Biosciences, Beverly, MA, USA), 2.5 µl of a 1 µM fluorescein disodium salt (VWR Life Science, PA, USA), 2.5 µl of primer mixture for Chr21 and Chr18 (Generi Biotech, Hradec Kralove, Czech Republic), with a concentration of 3.125 µM for each primer, 0.9 µl of a 6.25 µM FAM-labeled LNA probe (Integrated DNA Technologies, Leuven, Belgium) for detection of Chr21 amplicons, 0.4 µl of a 6.25 µM VIC-labeled LNA probe (Integrated DNA Technologies, Leuven, Belgium) for detection of Chr18 amplicons, 0.2 µl of deionized water, and 6 µl of the DNA sample.

### Digital droplet PCR

For ddPCR, Crystal Digital PCR developed by Naica System (Stilla Technologies, Villejuif, France) was used. Three sapphire chips were employed for each sample, allowing for four parallel tests for a total of 12 tests per sample. A total of 25 µl of the reaction mixture were pipetted into each position on the chip. Afterward, the chips were placed into the Naica Geode, where up to 25,000 droplets for each test were generated. The volume of each droplet was approximately 0.59 nl, as declared by the manufacturer.

Thermal cycling was performed on the same instrument. The following dPCR cycling conditions were applied: initially, 95 °C for 5 min, followed by a two-stage touchdown PCR consisting of 35 cycles of 95 °C for 30 s, 63 °C for 90 s, then 15 cycles of 95 °C for 30 s, and 56 °C for 90 s. The higher annealing temperature in the first part maintains the high specificity of the product formation, and the lower one in the second part allows for the accumulation of amplicons^27^.

### Result interpretation

The Naica Prism 3 (Stilla Technologies, Villejuif, France) reader was used to perform image acquisition with the following exposure times: blue channel 30 ms, green channel 50 ms, and red channel 1 ms. The total number of droplets and droplet quality control was performed by Crystal Reader software (Stilla Technologies, Villejuif, France). Fluorescein was used as the reference dye. Fluorescence values for each droplet were analyzed using Crystal Miner software.

Thresholds were manually set for all 12 tests of each sample. The results were reported in copies of target sequences per microliter of sample. All values obtained from each analyzed sample's 12 tests were combined, and the average value for both chromosomes was calculated. The ratio of Chr21 to Chr18 was computed from these merged values. This approach was repeated three times for each sample to avoid subjective mistakes in measurement. The final ratio was determined by averaging the results of these three measurements.

As a result of combining twelve tests for each sample, greater experimental precision was obtained, resulting in a coefficient of variation of less than 1%. The theoretical precision of ddPCR improves as the number of droplets increases (when performed within the optimal window of the dynamic range)^20^. Uncertainty has been identified as the most important quality control criterion.

### Statistical analysis

For statistical analysis, we used OriginPro (Origin Lab Corporation, USA).

### Supplementary Information

Supplementary Information 1.

Supplementary Information 2.

Supplementary Information 3.

## Data Availability

Data are available in the form of Supplementary data files.
