# Supplementary material for: Rapid non-invasive prenatal screening test for trisomy 21 based on digital droplet PCR
Source: Sci Rep. 2023 Dec 22;13:22948. doi: 10.1038/s41598-023-50330-x (PMC10746715; doi:10.1038/s41598-023-50330-x)
Supplement: Supplementary file 1 — Supplementary Information 1. [file 41598_2023_50330_MOESM1_ESM.docx]

**Supplementary Table 1. Information of gene locations and sequences of primer pairs for amplification of sequences from the chromosome 21 according to Tan et al. (2019).16 primer pairs were selected during multiplexddPCR optimization)**

|  | Gene location^1^ | Primer sequence (5’-3’) | Amplicon length (bp) |
| --- | --- | --- | --- |
| C | 16912895-16912966 | F: gcttctgggatgacacacg | 72 |
|  |  | R: cacatgatattgtgaaaagcaagtt |  |
| D | 18597570-18597636 | F: tgaattagttgcaaggtggttct | 67 |
|  |  | R: ccgagaagaagaaggattgaca |  |
| F | 24207756-24207825 | F: cataattcccactgtggtggtat | 70 |
|  |  | R: gcagagcccccatgaatta |  |
| G | 25698963-25699026 | F: agagctgataccattgcttgc | 64 |
|  |  | R: aagtgctgccatctttcctg |  |
| H | 25988208-25988278 | F: ggccagagctaaagaagcaa | 71 |
|  |  | R: ctgtacatgatgtcccccatt |  |
| I | 26492402-26492463 | F: tgagggaaatagaggtgctca | 62 |
|  |  | R: tgtgtcattgtttgccaattc |  |
| J | 27800999-27801086 | F: acgccaccatgcctagtta | 88 |
|  |  | R: tgaggtcgggaattcaagg |  |
| K | 29174833-29174909 | F: tgtgtggaacaactggagaaa | 77 |
|  |  | R: cactgaaaaatttaaagttaggacga |  |
| L | 31033224-31033289 | F: tcccttagcctaggttatctagca | 66 |
|  |  | R: ccatcaagcattagagacaaagg |  |
| M | 31509622-31509681 | F: gcatcaaggacccacagag | 60 |
|  |  | R: cagggcattggaactgtctt |  |
| N | 36205063-36205124 | F: acagggtgctgttgtcactg | 62 |
|  |  | R: agcaccgctttggtacaatc |  |
| O | 37959129-37959206 | F: agacttcttcgagaattgtgtaattg | 78 |
|  |  | R: aattgatgtctgtttgagttgatttta |  |
| P | 41284470-41284545 | F: ccactgaaattattctaactagccaaa | 76 |
|  |  | R: tgtggtttctgtaggaaagaagg |  |
| R | 42923863-42923928 | F: tgggtgaggtactgaaactctct | 66 |
|  |  | R: caaaagactcggtttatttccag |  |
| T | 44441621-44441686 | F: gcagtccgtagggctcag | 66 |
|  |  | R: agaacaagggcacagctgac |  |
| U | 45098547-45098621 | F: ccctggaacaaaactgttgag | 75 |
|  |  | R: gaaactgaggcccagcact |  |

**Supplementary Table 2. Information of gene locations and sequences of primer pairs for amplification of sequences from the chromosome 18 according to Tan et al. (2019) .16 primer pairs were selected during multiplexddPCR optimization)**

|  | Gene location1 | Primer sequence (5’-3’) | Amplicon length (bp) |
| --- | --- | --- | --- |
| A | 1248496-1248575 | F: ccatctccataacccaaatacc | 80 |
|  |  | R: ccttgcaaacctcatgttga |  |
| B | 5147229-5147324 | F: aatgaccaagaagccaccat | 96 |
|  |  | R: gcagttgccttaaacaaggtatatta |  |
| C | 8473597-8473674 | F: gagaaacctgcccacaagat | 78 |
|  |  | R: tctcctgttcggttataatgtcc |  |
| D | 8870993-8871084 | F: cctgtttgttctttaatagcctcat | 92 |
|  |  | R: tggcaaatttcaatgatgtcac |  |
| E | 13150636-13150721 | F: gcatcatcggatggttttg | 86 |
|  |  | R: ccttgctacttctaggctaacca |  |
| G | 23485174-23485251 | F: ggagcaaagggaaaacagg | 78 |
|  |  | R: tgccaattaaacatgctagacaa |  |
| H | 24943949-24944019 | F: tgaccagaggagcctggtag | 71 |
|  |  | R: ttcatcttcaaagagctccaga |  |
| J | 37782135-37782224 | F: gggtctccactctgatgatttg | 90 |
|  |  | R: tgggaacttaatcccaaaattaac |  |
| K | 48190141-48190240 | F: tgggagtgcagtgtctgtct | 100 |
|  |  | R: acacttagtcttcttcttgctcctg |  |
| M | 50208031-50208106 | F: aacttgcaaggttttcactgg | 76 |
|  |  | R: tgggccatgctgtattcat |  |
| N | 51129532-51129592 | F: tgagctcagggtggaaagag | 61 |
|  |  | R: caagggattacgcatgcac |  |
| O | 51295343-51295441 | F: catggttctgcaggctatacaa | 99 |
|  |  | R: ttgtcccctccaaatgtcag |  |
| P | 51951491-51951550 | F: aagacaggagagcgaggtga | 60 |
|  |  | R: ttctgttaataaggcccatgc |  |
| Q | 52280540-52280623 | F: aaacattggaatcagactgaggtag | 84 |
|  |  | R: agaagttacaacttacccacgctta |  |
| R | 56126533-56126611 | F: ccctcacatcccttccaac | 79 |
|  |  | R: ccctgcattaacccctcag |  |
| S | 56554079-56554149 | F: tttctgactctgcaatctgctta | 71 |
|  |  | R: cacagaggtgaaggcacaaa |  |

^1^ Gene location refers to the human genome assembly version hg19/Genome Reference Consortium Human Build 37 (GRCh37)

**Supplementary Table 3. Information of chromosome locations and sequences of probes according to Tan et al. (2019)**

| Chromosome | Probe sequence (5’-3’)^2^ | Probe length (nt) |
| --- | --- | --- |
| 21 | 5'-FAM/C+C+CT+G+CCT+CT/3'-IABkFQ | 10 |
| 18 | 5'-YAkYel/C+C+CA+C+CTC+CA/3'- IABkFQ | 10 |

^2^ plus sign “+” represents locked nucleic acid (LNA)

**Supplementary Table 4. Reasons for exclusion primer pairs during multiplex ddPCR optimization**

| Reason for exclusion | Cause | Primer |
| --- | --- | --- |
| I. | no amplification of a target | 21 E |
| II. | amplification with the primer pair led to higher amount of product than with the other primer pairs | 21 A |
| III. | the same amplicon was detected by both probes | 21 B |
|  |  | 18 I |
| IV. | amplification with the primer pair was successful but elevated the fluorescence of negative droplets in the second channel | 18 F |
|  |  | 18 L |
|  |  | 18 T |

Primer 21 Q was excluded in order to exclude the same number of primers for both chromosomes. Primer 21 Q caused more ,,rain“ compared to other primers for chromosome 21.

Reference:

Tan C, Chen X, Wang F, Wang D, Cao Z, Zhu X, et al. A multiplex droplet digital PCR assay for non-invasive prenatal testing of fetal aneuploidies. The Analyst. 2019;144:2239–47.
