# Supplementary material for: Rapid non-invasive prenatal screening test for trisomy 21 based on digital droplet PCR
Source: Sci Rep. 2023 Dec 22;13:22948. doi: 10.1038/s41598-023-50330-x (PMC10746715; doi:10.1038/s41598-023-50330-x)
Supplement: Supplementary file 2 — Supplementary Information 2. [file 41598_2023_50330_MOESM2_ESM.docx]

**Supplementary Table 5.** 21/18 values (ratios of Chr 21 to Chr 18) obtained in the analysis of artificial mixtures, each mixture was analyzed in 12 technical replicates and values of uncertainity reported before and after merging.

| Replicate No. | 0 % of T21 | Uncertainity for | | 5 % of T21 | Uncertainity for | | 10% of T21 | Uncertainity for | | 15% of T21 | Uncertainity for | | 20% of T21 | Uncertainity for | |
| --- | --- | --- | --- | --- | --- | --- | --- | --- | --- | --- | --- | --- | --- | --- | --- |
|  | **21/18** | **Chr 21(%)** | **Chr 18(%)** | **21/18** | **Chr 21(%)** | **Chr 18(%)** | **21/18** | **Chr 21(%)** | **Chr 18(%)** | **21/18** | **Chr 21(%)** | **Chr 18(%)** | **21/18** | **Chr 21(%)** | **Chr 18(%)** |
| 1 | 0.9846 | 4.11 | 4.08 | 1.0022 | 4.47 | 4.47 | 0.9801 | 3.93 | 3.93 | 0.9640 | 4.65 | 4.56 | 1.0660 | 6.04 | 6.23 |
| 2 | 0.9735 | 4.15 | 4.09 | 0.9675 | 4.15 | 4.09 | 0.9743 | 3.93 | 3.93 | 0.9682 | 4.35 | 4.29 | 1.0227 | 4.59 | 4.64 |
| 3 | 0.9896 | 4.09 | 4.07 | 0.9667 | 4.16 | 4.09 | 1.0358 | 3.94 | 3.94 | 1.0327 | 4.20 | 4.27 | 1.0642 | 4.40 | 4.53 |
| 4 | 0.9505 | 4.16 | 4.06 | 0.9965 | 3.97 | 3.96 | 0.9327 | 3.91 | 3.91 | 0.9637 | 4.36 | 4.28 | 1.0113 | 4.42 | 4.45 |
| 5 | 0.9853 | 4.35 | 4.32 | 1.0172 | 4.04 | 4.08 | 1.0126 | 4.00 | 4.00 | 1.0346 | 4.22 | 4.29 | 1.0881 | 4.67 | 4.86 |
| 6 | 0.9679 | 4.25 | 4.19 | 0.9724 | 4.05 | 4.00 | 1.0740 | 4.30 | 4.30 | 1.0686 | 4.19 | 4.32 | 1.1200 | 4.58 | 4.84 |
| 7 | 0.9732 | 4.09 | 4.04 | 0.9708 | 4.11 | 4.05 | 1.0329 | 4.00 | 4.00 | 1.0387 | 4.17 | 4.25 | 1.0592 | 4.37 | 4.49 |
| 8 | 0.9778 | 4.07 | 4.03 | 1.0068 | 4.07 | 4.08 | 0.9942 | 4.00 | 4.00 | 1.0501 | 4.15 | 4.25 | 1.1534 | 4.28 | 4.58 |
| 9 | 0.9165 | 4.37 | 4.19 | 0.9965 | 4.10 | 4.09 | 1.0477 | 4.00 | 4.00 | 1.0588 | 4.34 | 4.46 | 1.1469 | 4.90 | 5.23 |
| 10 | 0.9852 | 4.14 | 4.11 | 0.9702 | 4.06 | 4.00 | 1.0279 | 3.99 | 3.99 | 0.9984 | 4.28 | 4.27 | 1.0777 | 4.73 | 4.91 |
| 11 | 0.9813 | 4.18 | 4.14 | 0.9766 | 4.02 | 3.97 | 1.0698 | 4.02 | 4.02 | 1.0967 | 4.17 | 4.36 | 1.1226 | 4.48 | 4.74 |
| 12 | 0.9762 | 4.10 | 4.06 | 1.0013 | 3.98 | 3.98 | 1.0268 | 3.94 | 3.94 | 1.0545 | 4.22 | 4.32 | 1.1050 | 4.32 | 4.54 |
| R values and uncertainity after merging | **0.9718** | **1.21** | **1.22** | **0.9871** | **1.22** | **1.22** | **1.0174** | **1.18** | **1.19** | **1.0274** | **1.26** | **1.28** | **1.0864** | **1.36** | **1.42** |
