# Supplementary material for: Rapid non-invasive prenatal screening test for trisomy 21 based on digital droplet PCR
Source: Sci Rep. 2023 Dec 22;13:22948. doi: 10.1038/s41598-023-50330-x (PMC10746715; doi:10.1038/s41598-023-50330-x)
Supplement: Supplementary file 3 — Supplementary Information 3. [file 41598_2023_50330_MOESM3_ESM.docx]

|  | First measurement | | | Second measurement | | | | Third measurement | | |  |
| --- | --- | --- | --- | --- | --- | --- | --- | --- | --- | --- | --- |
| Sample | **Chr 21** | **Chr18** | **Chr21/Chr18** | **Chr 21** | **Chr18** | **Chr21/Chr18** | | **Chr 21** | **Chr18** | **Chr21/Chr18** | **Average** |
| 1 | 1932 | 1797 | 1.075125 | 1943 | 1806 | 1.075858 | | 1932 | 1796 | 1.075858 | 1.0756 |
| 2 | 497.6 | 472.2 | 1.053791 | 498.3 | 473.5 | 1.052376 | | 495.9 | 472 | 1.052376 | 1.0528 |
| 3 | 975 | 930.6 | 1.047711 | 978 | 932.9 | 1.048344 | | 978.5 | 933 | 1.048344 | 1.0481 |
| 4 | 449 | 414 | 1.084541 | 449.3 | 414.7 | 1.083434 | | 448.7 | 413.9 | 1.083434 | 1.0838 |
| 5 | 549 | 504.8 | 1.087559 | 549.6 | 506.2 | 1.085737 | | 549.9 | 506.3 | 1.085737 | 1.0863 |
| 6 | 512.3 | 476.5 | 1.075131 | 510.1 | 477.1 | 1.069168 | | 510.4 | 475.7 | 1.069168 | 1.0712 |
| 7 | 831.6 | 775.7 | 1.072064 | 831.4 | 776.3 | 1.070978 | | 833.2 | 776.2 | 1.070978 | 1.0713 |
| 8 | 349.1 | 330.1 | 1.057558 | 347.1 | 329.8 | 1.052456 | | 348.9 | 330.2 | 1.052456 | 1.0542 |
| 9 | 561.1 | 526.3 | 1.066122 | 562.5 | 525.7 | 1.070002 | | 563.3 | 526.1 | 1.070002 | 1.0687 |
| 10 | 348.9 | 320.4 | 1.088951 | 348.4 | 320.1 | 1.088410 | | 349.8 | 320.7 | 1.088410 | 1.0886 |
| 11 | 699.1 | 632.4 | 1.105471 | 698.9 | 634.9 | 1.100803 | | 700.3 | 633.8 | 1.100803 | 1.1024 |
| 12 | 286.1 | 276.1 | 1.036219 | 294.5 | 282.4 | 1.042847 | | 292.1 | 278.9 | 1.042847 | 1.0406 |
| 13 | 373.5 | 349.8 | 1.067753 | 370.3 | 350.2 | 1.057396 | | 374.5 | 350 | 1.057396 | 1.0608 |
| 14 | 451.9 | 421.4 | 1.072378 | 455.5 | 423 | 1.076832 | | 454.6 | 422.7 | 1.076832 | 1.0753 |
| 15 | 389.3 | 359.8 | 1.08199 | 393.8 | 362.2 | 1.087245 | | 393 | 361.1 | 1.087245 | 1.0855 |
| 16 | 481.9 | 458.4 | 1.051265 | 486.9 | 460.3 | 1.057788 | | 484.2 | 459.2 | 1.057788 | 1.0556 |
| 17 | 430 | 404.1 | 1.064093 | 433.4 | 408.5 | 1.060955 | | 431.7 | 405.2 | 1.060955 | 1.0620 |
| 18 | 841 | 770.1 | 1.092066 | 844.8 | 772.5 | 1.093592 | | 845.9 | 771 | 1.093592 | 1.0931 |
| 19 | 821.2 | 766.5 | 1.071363 | 828.7 | 769.2 | 1.077353 | | 827.1 | 767.1 | 1.077353 | 1.0754 |
| 20 | 829.2 | 792.2 | 1.046705 | 830.5 | 794.5 | 1.045312 | | 827.9 | 792.4 | 1.045312 | 1.0458 |
| 21 | 606.5 | 562.7 | 1.077839 | 611.8 | 565.9 | 1.081110 | | 608.8 | 564.7 | 1.081110 | 1.0800 |
| 22 | 645.5 | 596.2 | 1.08269 | 649.4 | 602.5 | 1.077842 | | 644 | 595.2 | 1.077842 | 1.0795 |
| 23 | 1187 | 1142 | 1.039405 | 1194 | 1148 | 1.040070 | | 1190 | 1145 | 1.040070 | 1.0398 |
| 24 | 772.1 | 718.1 | 1.075198 | 780 | 726.5 | 1.073641 | | 771.3 | 719.8 | 1.073641 | 1.0742 |
| 25 | 641 | 600.9 | 1.066733 | 644.5 | 604.7 | 1.065818 | | 640.3 | 601.4 | 1.065818 | 1.0661 |
| 26 | 542.8 | 500.7 | 1.084082 | 546.6 | 504.2 | 1.084094 | | 544.6 | 500.7 | 1.084094 | 1.0841 |
| Average | | | | | | | **1.0700** | | | | |
| SD | | | | | | | **0.0166** | | | | |

**Supplementary Table 6.** Results of multiplex ddPCR for control group (cp/ul)

|  | First measurement | | | Second measurement | | | | Third measurement | | |  |
| --- | --- | --- | --- | --- | --- | --- | --- | --- | --- | --- | --- |
| Sample | **Chr 21** | **Chr18** | **Chr21/Chr18** | **Chr 21** | **Chr18** | | **Chr21/Chr18** | **Chr 21** | **Chr18** | **Chr21/Chr18** | **Average** |
| 1 | 1018 | 876.1 | 1.161968 | 1016 | 875.8 | | 1.160082 | 1013 | 874.7 | 1.158111 | 1.1601 |
| 2 | 772.2 | 715.2 | 1.079698 | 770.5 | 714.8 | | 1.077924 | 769.6 | 714.1 | 1.077720 | 1.0784 |
| 3 | 834.4 | 739 | 1.129093 | 830.7 | 735.9 | | 1.128822 | 824.9 | 735.8 | 1.121093 | 1.1263 |
| 4 | 434.5 | 381.8 | 1.13803 | 433.6 | 380.7 | | 1.138955 | 432.3 | 381.3 | 1.133753 | 1.1369 |
| 5 | 499.5 | 454.9 | 1.098044 | 497.8 | 453.7 | | 1.097201 | 496.6 | 452.1 | 1.098430 | 1.0979 |
| 6 | 325 | 293.9 | 1.105818 | 322.3 | 292.3 | | 1.102634 | 322.2 | 292.7 | 1.100786 | 1.1031 |
| 7 | 1408 | 1264 | 1.113924 | 1406 | 1259 | | 1.116759 | 1406 | 1259 | 1.116759 | 1.1158 |
| 8 | 1102 | 918.7 | 1.199521 | 1100 | 914 | | 1.203501 | 1092 | 915.4 | 1.192921 | 1.1986 |
| 9 | 541.8 | 491.9 | 1.101443 | 540.8 | 490.2 | | 1.103223 | 539.8 | 490 | 1.101633 | 1.1021 |
| 10 | 652.2 | 613 | 1.063948 | 649.8 | 611.4 | | 1.062807 | 641.3 | 611.4 | 1.048904 | 1.0586 |
| 11 | 516.3 | 456.6 | 1.130749 | 514.4 | 455.4 | | 1.129556 | 513.4 | 455.6 | 1.126866 | 1.1291 |
| 12 | 559.7 | 482.2 | 1.160722 | 558.1 | 481.2 | | 1.159809 | 552.4 | 481 | 1.148441 | 1.1563 |
| 13 | 735.7 | 658.3 | 1.117576 | 733.5 | 656.6 | | 1.117118 | 733.3 | 655.5 | 1.118688 | 1.1178 |
| 14 | 346.3 | 304.6 | 1.136901 | 345.5 | 302.6 | | 1.141771 | 342.9 | 303.2 | 1.130937 | 1.1365 |
| 15 | 796.6 | 671.7 | 1.185946 | 790.4 | 665.6 | | 1.1875 | 799.8 | 688.3 | 1.161993 | 1.1785 |
| 16 | 771.2 | 680.5 | 1.133284 | 763.7 | 672.3 | | 1.135951 | 766 | 672.7 | 1.138695 | 1.1360 |
| Average | | | | | | **1.1270** | | | | | |
| SD | | | | | | **0.0359** | | | | | |

**Supplementary Table 7.** Results of multiplex ddPCR for T21 group (cp/ul)

**Supplementary Table 8.** Results of multiplex ddPCR for validation group (cp/ul)

|  | First measurement | | | Second measurement | | | Third measurement | | |  |
| --- | --- | --- | --- | --- | --- | --- | --- | --- | --- | --- |
| Sample | **Chr 21** | **Chr18** | **Chr21/Chr18** | **Chr 21** | **Chr18** | **Chr21/Chr18** | **Chr 21** | **Chr18** | **Chr21/Chr18** | **Average** |
| 1 | 1009 | 869.2 | 1.160838 | 1006 | 867.5 | 1.159654 | 1006 | 866.6 | 1.160859 | 1.1605 |
| 2 | 693.6 | 603.9 | 1.148535 | 693.8 | 604.6 | 1.147536 | 693.2 | 604.5 | 1.146733 | 1.1476 |
| 3 | 580.5 | 507.3 | 1.144293 | 580.5 | 506.4 | 1.146327 | 580.1 | 504.1 | 1.150764 | 1.1471 |
| 4 | 1335 | 1162 | 1.148881 | 1335 | 1161 | 1.149871 | 1334 | 1161 | 1.149009 | 1.1493 |
| 5 | 602.9 | 541.9 | 1.112567 | 598.9 | 540.6 | 1.107843 | 601.8 | 541.7 | 1.110947 | 1.1105 |
| 6 | 843.5 | 759.3 | 1.110892 | 842.9 | 758.8 | 1.110833 | 842.7 | 758.2 | 1.111448 | 1.1111 |
| 7 | 308.4 | 296.6 | 1.039784 | 308 | 296.6 | 1.038436 | 307.9 | 296.6 | 1.038098 | 1.0388 |
| 8 | 495.5 | 457.9 | 1.082114 | 495.7 | 457.1 | 1.084445 | 495.6 | 456.8 | 1.084939 | 1.0838 |
| 9 | 455.9 | 419.7 | 1.086252 | 454.4 | 415.9 | 1.092570 | 453.6 | 415.9 | 1.090647 | 1.0898 |
| 10 | 1046 | 962.3 | 1.086979 | 1047 | 963.1 | 1.087115 | 1046 | 962.6 | 1.086640 | 1.0869 |
| 11 | 451.5 | 412.2 | 1.095342 | 450.2 | 411.5 | 1.094046 | 452.5 | 413.8 | 1.092798 | 1.0941 |
| 12 | 1001 | 915.6 | 1.093272 | 1001 | 916.2 | 1.092556 | 997.4 | 914 | 1.091247 | 1.0924 |
| 13 | 795.4 | 729.5 | 1.090336 | 795 | 729.4 | 1.089937 | 794.7 | 729.6 | 1.089227 | 1.0898 |
| 14 | 880.8 | 824.9 | 1.067766 | 879.7 | 824.4 | 1.067079 | 880.2 | 824.5 | 1.067556 | 1.0675 |
| 15 | 473.5 | 450.1 | 1.051988 | 473.1 | 449.9 | 1.051567 | 472.9 | 449.9 | 1.051122 | 1.0516 |
| 16 | 590.7 | 550.9 | 1.072245 | 590.8 | 551.5 | 1.071260 | 590.8 | 550.9 | 1.072427 | 1.0720 |
| 17 | 1866 | 1733 | 1.076746 | 1864 | 1733 | 1.075591 | 1866 | 1733 | 1.076746 | 1.0764 |
| 18 | 426.4 | 392.1 | 1.087478 | 426.1 | 392.3 | 1.086159 | 426.5 | 392.6 | 1.086347 | 1.0867 |
| 19 | 123.8 | 114.6 | 1.080279 | 123.8 | 114.6 | 1.080279 | 123.9 | 114.6 | 1.081152 | 1.0806 |
| 20 | 683.2 | 638.4 | 1.070175 | 680.7 | 635.7 | 1.070788 | 681.2 | 635.9 | 1.071238 | 1.0707 |
| 21 | 321.5 | 295.6 | 1.087618 | 320.6 | 294.6 | 1.088255 | 320.9 | 294.8 | 1.088535 | 1.0881 |
| 22 | 462.1 | 437.5 | 1.056229 | 462.1 | 437.5 | 1.056229 | 462.1 | 437.4 | 1.056470 | 1.0563 |
| 23 | 754.7 | 707 | 1.067468 | 752.4 | 706.8 | 1.064516 | 752.7 | 706.5 | 1.065393 | 1.0658 |
| 24 | 533.1 | 498.1 | 1.070267 | 531.7 | 497.7 | 1.068314 | 533.5 | 497.7 | 1.071931 | 1.0702 |
| 25 | 320.4 | 309.1 | 1.036558 | 320.3 | 308.8 | 1.037241 | 320.1 | 308.8 | 1.036593 | 1.0368 |
| 26 | 822.5 | 751.2 | 1.094915 | 820.7 | 750.6 | 1.093392 | 821.9 | 751.1 | 1.094262 | 1.0942 |
| 27 | 531.3 | 491.4 | 1.081197 | 530.9 | 491.4 | 1.080383 | 531 | 491 | 1.081466 | 1.0810 |
| 28 | 508.3 | 464.5 | 1.094295 | 510.9 | 466.4 | 1.095412 | 507.9 | 464.5 | 1.093434 | 1.0944 |
| 29 | 420.6 | 392.3 | 1.072139 | 420.7 | 392.1 | 1.072941 | 420.5 | 392.3 | 1.071884 | 1.0723 |
| 30 | 1841 | 1753 | 1.0502 | 1842 | 1753 | 1.05077 | 1842 | 1751 | 1.05197 | 1.0510 |
